# Identification of a Novel Subset of Human Airway Epithelial Basal Stem Cells

**DOI:** 10.3390/ijms25189863

**Published:** 2024-09-12

**Authors:** Christopher Cheng, Parul Katoch, Yong-Ping Zhong, Claire T. Higgins, Maria Moredock, Matthew E. K. Chang, Mark R. Flory, Scott H. Randell, Philip R. Streeter

**Affiliations:** 1 Oregon Stem Cell Center, Papè Family Pediatric Research Institute, Department of Pediatrics, Oregon Health and Science University, Portland, OR 97239-3098, USA; 2 Cancer Early Detection Advanced Research Center, Knight Cancer Institute, Oregon Health and Science University, Portland, OR 97239-3098, USA; 3 Marsico Lung Institute/Cystic Fibrosis Research Center, The University of North Carolina at Chapel Hill, Chapel Hill, NC 27599-7248, USA

**Keywords:** monoclonal antibody, stem cells, basal cells, cell heterogeneity, respiratory epithelium, bronchospheres, aminooxy-sulfhydryl-biotin crosslinking, CD71, glycosylated transferrin receptor, fluorescence activated cell sorting

## Abstract

The basal cell maintains the airway’s respiratory epithelium as the putative resident stem cell. Basal cells are known to self-renew and differentiate into airway ciliated and secretory cells. However, it is not clear if every basal cell functions as a stem cell. To address functional heterogeneity amongst the basal cell population, we developed a novel monoclonal antibody, HLO1-6H5, that identifies a subset of KRT5+ (cytokeratin 5) basal cells. We used HLO1-6H5 and other known basal cell-reactive reagents to isolate viable airway subsets from primary human airway epithelium by Fluorescence Activated Cell Sorting. Isolated primary cell subsets were assessed for the stem cell capabilities of self-renewal and differentiation in the bronchosphere assay, which revealed that bipotent stem cells were, at minimum 3-fold enriched in the HLO1-6H5+ cell subset. Crosslinking-mass spectrometry identified the HLO1-6H5 target as a glycosylated TFRC/CD71 (transferrin receptor) proteoform. The HLO1-6H5 antibody provides a valuable new tool for identifying and isolating a subset of primary human airway basal cells that are substantially enriched for bipotent stem/progenitor cells and reveals TFRC as a defining surface marker for this novel cell subset.

## 1. Introduction

The human respiratory system can be divided into two regions based on function: the gas-exchange alveolar space and the conducting airways comprising the trachea, bronchi, and bronchioles. The basal cell population maintains the airway’s pseudostratified respiratory epithelium, which is composed of columnar ciliated and secretory cells that are apically exposed to the lumen and basal cells that reside along the basement membrane away from the lumen. Basal cells are the stem cell population of the airways, capable of both self-renewal and differentiation into the secretory and ciliated cell lineages [1,2,3,4,5]. These stem cell properties make the basal cell population a focus of lung regeneration and engineering studies. Recent studies with uncultured primary tissue have revealed that the basal cell compartment is heterogeneous, with subpopulations of basal cells of varying stem/progenitor cell capabilities [6,7,8]. Functional studies of potential airway stem cell populations have been difficult due to the dearth of molecular tools that can target cell surface markers to isolate viable cells. Hence, a critical need exists for the development of new tools to enable the study of basal cell subsets [9,10,11].

The set of molecular markers defining lung basal cells has, to date, remained relatively limited. The airway basal cell is phenotypically defined by the expression of cytokeratin 5 (KRT5) and tumor protein p63, with a mitotically active subset expressing cytokeratin 14 (KRT14) [10,12,13]. Nerve growth factor receptor (NGFR) and integrin alpha-6 (ITGA6) are cell surface markers enriched in airway basal cells and used to identify and isolate bulk basal cells from primary and cultured human airway tissues for study in a functional bronchosphere assay [3,14,15,16]. Several human and mouse studies have identified phenotypically and functionally distinct subsets of basal cells using single-cell RNA sequencing and lineage tracing [6,7,8,17,18,19]. Unfortunately, most of those studies identified basal cell subsets by intracellular markers, which yield non-viable cells that preclude subsequent functional studies. New tools to target surface markers on basal cells will enable functional studies on population heterogeneity [12,13,20].

In the work described here, we identify a novel subset of human basal cells using a monoclonal antibody (mAb) reagent generated following immunizations with intact human lung organoids. The HLO1-6H5 mAb specifically identifies a subset of KRT5+ basal cells in primary human airway epithelium. To further characterize the cellular reactivity of this antibody, we employed fluorescence-activated cell sorting (FACS) to isolate HLO1-6H5-positive basal cell subsets from conducting airway epithelium and assessed the isolated cells for self-renewal/proliferation and differentiation potential in an in vitro bronchosphere assay [3,21,22,23]. This study revealed that the HLO1-6H5-positive cells are a novel subset of airway basal cells, and that this subset is highly enriched for bipotent stem/progenitor cells. Crosslinking-mass spectrometry data indicate that the HLO1-6H5 mAb binds with a glycosylated transferrin receptor (TFRC/CD71) proteoform.

## 2. Results

### 2.1. Generation of a Novel Monoclonal Antibody for Human Airway Basal Cells

**Figure 1:**
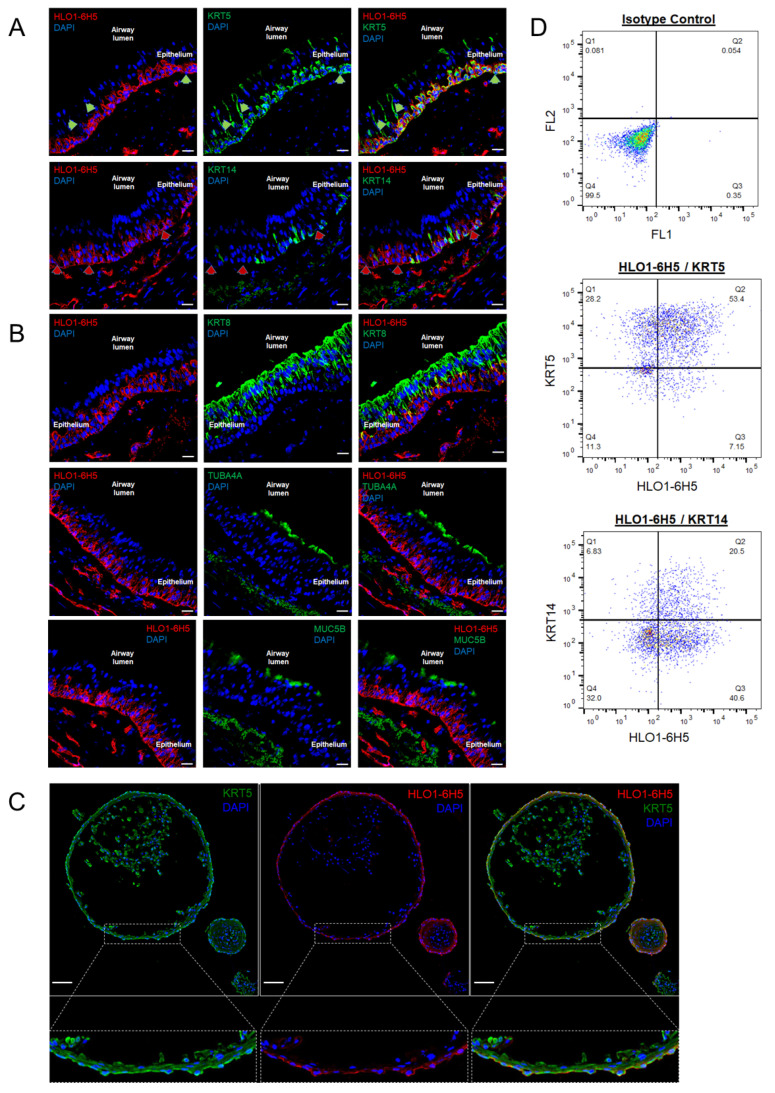
HLO1-6H5 identifies a subset of human basal cells. Representative immunohistochemical co-staining of human proximal airway with the HLO1-6H5 monoclonal antibody (mAb), in combination with canonical markers, that identify specific airway epithelial cell types such as (**A**) basal cells (KRT5, cytokeratin 5) and mitotically active basal cells (KRT14, cytokeratin 14). Green arrows indicate basal cells that are KRT5+/HLO1-6H5−. Red arrows indicate HLO1-6H5+ cells along the basal laminae that are KRT14-. HLO1-6H5 were also co-stained with markers for (**B**) luminal columnar cells (KRT8, cytokeratin 8), such as ciliated cells (TUBA4A, acetylated-alpha tubulin) and secretory cells (Muc5B, mucin 5B). (n = three donor samples) Scale bar: 20 µm. (**C**) Day 28 bronchospheres derived from human bronchial epithelial cells (hBECs) are also stained for basal cells (KRT5) and HLO1-6H5+ basal cells. Scale bar: 200 µm. (**D**) Representative flow cytometry plots of HLO1-6H5 co-expression with KRT5 or KRT14 in uncultured primary hBECs. (n = three donor samples).

Novel mouse anti-human mAbs were generated by immunizing mice with intact lung organoids, a source of enriched stem/progenitor cells, grown from primary human bronchial epithelial cells (hBECs). Hybridomas were generated using standard cell–cell fusion methods [24,25,26]. Hybridomas were screened by immunohistochemistry (IHC) on human airways and by flow cytometric analysis on viable uncultured primary hBECs. IHC staining showed that HLO1-6H5 targeted cells in the basal compartment of respiratory epithelium (Figure 1A,B).

Flow cytometric analyses with HLO1-6H5 mAb revealed selective targeting of a cell-surface epitope on an epithelial cell subset (Figure 1D). HLO1-6H5 was determined to be an immunoglobulin M (IgM) isotype.

### 2.2. The Novel HLO1-6H5 Monoclonal Antibody Targets a Subset of Basal Cells in Human Proximal Airway Epithelium

Immunohistochemistry (IHC) showed that the HLO1-6H5 mAb targeted an antigen expressed on basal cells, and not on ciliated or secretory cells in human airway epithelium (Figure 1A,B). The only cells that expressed the HLO1-6H5 target marker were located on the basement membrane and not exposed to the airway lumen. The HLO1-6H5 marker colocalized with a large subset of basal cells (KRT5+) in airway tissue and targeted the entire KRT14+ subset of basal cells (Figure 1A). HLO1-6H5+ cells did not co-express with the luminal differentiation markers cytokeratin 8 (KRT8), the ciliated cell marker acetylated-alpha tubulin (TUBA4A), or the secretory goblet cell marker mucin 5B (MUC5B) (Figure 1B). The HLO1-6H5 mAb also identified a subset of cells in the submucosa below the respiratory epithelium. Co-staining of airway tissue sections with HLO1-6H5 and anti-CD31 (endothelial cell marker) antibodies showed that HLO1-6H5 also identifies endothelial cells, however HLO1-6H5 targeting of endothelial cells did not impact hBEC analyses or sorting as the hBEC harvesting protocol in the primary airway excludes endothelial cells.

In an in vitro model of the human proximal airway, the HLO1-6H5 mAb also stained basal cells. In this model, 3-dimensional bronchospheres are derived from airway stem cells, with spheres containing multiple airway cell types. Immunocytochemistry (ICC) of bronchosphere sections showed that KRT5+ basal cells comprise most of the cells in the structure, but the HLO1-6H5 mAb identified only the subset of KRT5+ cells located on the exterior layer of the bronchosphere wall (Figure 1C).

The HLO1-6H5 pattern of targeting airway basal cells was maintained in uncultured hBECs from healthy non-smoker donor proximal airways (Table S1). Flow cytometry revealed uncultured primary hBECs are composed almost entirely of KRT5+ cells, indicating that most of the hBECs were basal cells across three independent adult donors. Of those KRT5+ primary hBECs, HLO1-6H5 was co-expressed only on a subset of them (Figure 1D). The HLO1-6H5+ subset was a 56–76% subset of total KRT5+ cells across the three donors (Figure 1D). The HLO1-6H5+ subset also contained a subset of KRT14+ cells ranging from 33–37% across the three donors (Figure 1D). Overall, the IHC and ICC staining indicates that the HLO1-6H5 mAb targets basal cells. Furthermore, the flow cytometric evidence shows that HLO1-6H5 labels a discrete subset of KRT5+ basal cells.

### 2.3. The Bronchosphere Assay Assesses Self-Renewal and Differentiation in Human Airway Epithelial Cells

**Figure 2:**
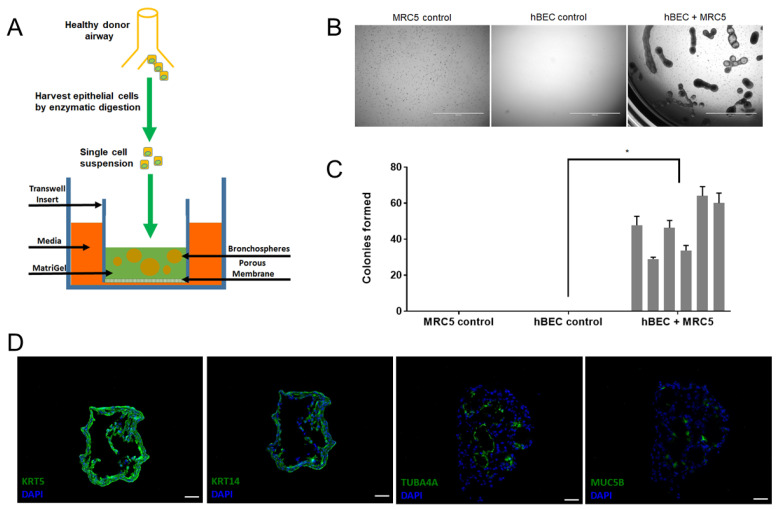
Primary hBECs require MRC5 stromal cells to form bronchospheres composed of differentiated airway epithelial cell lineages. (**A**) Primary hBECs are harvested from proximal airway and seeded as single cells into MatriGel^®^ matrix that is layered into a Transwell^®^ insert to generate an air–liquid interface culture condition. (**B**) Bronchospheres grown for 14 days from unsorted hBECs co-cultured with MRC5 human fetal lung fibroblasts. Scale bars, 2000 µm. (**C**) Bar graph of number of bronchospheres formed by MRC5 fibroblasts, hBECs, and hBECs co-cultured with MRC5 fibroblasts across six donors, three technical replicates per donor. Data presented as mean (±S.D.). Paired *t*-test performed, * *p* < 0.05. (**D**) Bronchospheres grown for 42 days from unsorted hBECs form lumens in the interior of the spheroid and contain basal cells that are KRT5+, mitotically active basal cells (KRT14+), ciliated cells (TUBA4A+), and secretory goblet cells (MUC5B+). Scale bar: 50 µm.

Primary hBECs from healthy non-smoker donor airways were embedded into the MatriGel^®^ matrix within a Transwell^®^ insert for growth in bronchosphere conditions to evaluate the stem cell properties of self-renewal and differentiation (Figure 2A and Table S1).

The bronchosphere assay from Rock et al.’s 2009 study used hBECs that are cultured overnight on collagen-coated plastic prior to input into the bronchosphere assay [3]. Our study focused on the identification of stem cell subsets that can be isolated from uncultured primary hBECs. These uncultured hBECs require support cells to form bronchospheres. The MRC5 stromal cell line is derived from the human fetal lung and has been used in human bronchosphere and alveolosphere assays, as a support cell, to increase colony-forming efficiency (CFE) [21,22,23]. In the absence of MRC5 cells, primary hBECs did not form bronchospheres, thereby preventing assessment of stem cell function (n = 6 donors) (Figure 2B,C). Adding MRC5 cells to the bronchosphere assay recovers colony-forming capabilities and enables assessment of stem cell properties.

Bronchosphere colonies are derived from single cells. Therefore, the assay assesses the stem cell property of self-renewal. To rule out any colony formation due to aggregation of multiple input cells, a mixture of GFP- (green fluorescent protein) and dsRed- (Discosoma red fluorescent protein) labeled hBECs were used to generate bronchospheres (Figure S1A). The colonies that formed were either homogenously GFP expressing, dsRed expressing, or not fluorescently labeled (Figure S1B). There were no bronchospheres of mixed GFP and dsRed expression, which would have been indicative of a colony that formed from more than one cell. Therefore, the bronchospheres were clonally derived (Figure S1B and Table S2).

The bronchosphere assay also assessed the stem cell property of differentiation or potency. Since each colony arose from a single cell, the emergence of cell types that were not present in the input population was indicative of cellular differentiation. Bronchospheres grown from primary hBECs were harvested after 42 days of culture when lumen formation was most evident. The colonies expressed markers of all three major airway epithelial cell types: basal, ciliated, and goblet (Figure 2D). All the colonies contained basal cells as evidenced by KRT5 and KRT14 expression (Figure 2D). Luminal cell lineages were evident in most of the bronchospheres that had lumens, as indicated by the expression of TUBA4A (acetylated-alpha tubulin) for ciliated cells, and MUC5B (mucin 5B) for goblet cells (Figure 2D). In summary, the bronchosphere assay revealed that the HLO1-6H5 cell subset exhibited properties of self-renewal and potency in primary hBECs.

### 2.4. Stem Cells of the Human Airway Express the HLO1-6H5 Marker

**Figure 3:**
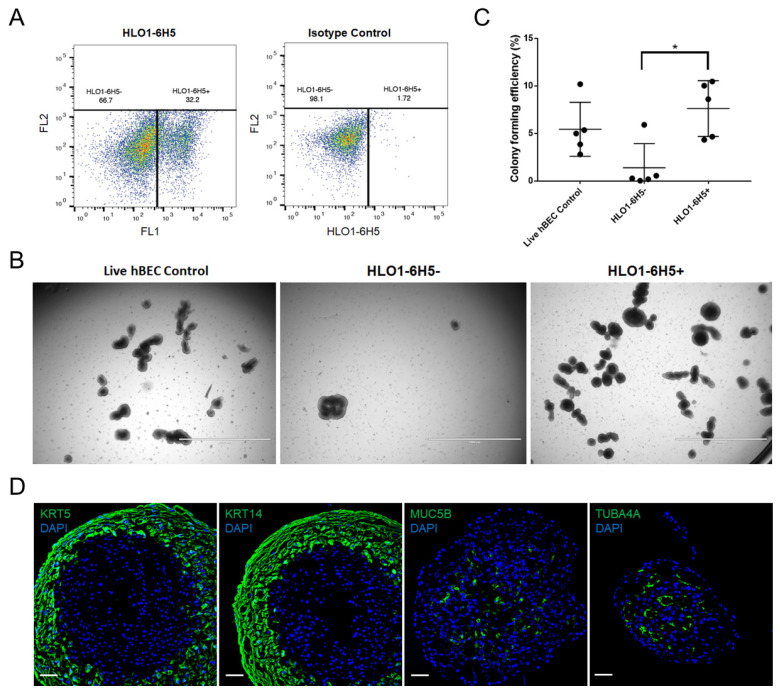
Fluorescence activated cell sorting (FACS) -isolated HLO1-6H5+ basal cells are functionally enriched for a bipotent stem/progenitor cell population. (**A**) Representative data from FACS-isolation of HLO1-6H5 subsets for assessment of clonogenicity and potency in the bronchosphere assay (n = 5 donor samples). (**B**) After 14 days of culture, colonies were counted to determine which HLO1-6H5 subset was enriched for clonogenic cells. Scale bar: 2000 µm. (**C**) Scatter plot shows the number of colonies formed as a percentage relative to the number of input cells (colony-forming efficiency). Paired *t*-test performed, * *p* < 0.05. Data presented as mean (±S.D.). (**D**) Bronchospheres derived from HLO1-6H5+ basal cells were sectioned and analyzed by immunocytochemistry to determine if the cellular composition of the colonies included basal cells (KRT5), mitotically active basal cells (KRT14), ciliated cells (TUBA4A), and/or secretory cells (MUC5B). Scale bar: 50 µm.

The HLO1-6H5 mAb was screened by flow cytometry and showed that the mAb labeled 25–65% of total hBECs across multiple donors, demonstrating that the marker is a cell surface antigen (Figure 3A) (n = 9).

Primary hBEC subsets were FACS-isolated with HLO1-6H5 mAb into the bronchosphere assay, and after 2 weeks of culture, colonies were evident, especially in the HLO1-6H5+ subset (Figure 3B). The HLO1-6H5 marker was positively enriched for clonogenic cells in the bronchosphere assay as there was a five-fold increase in CFE between the HLO1-6H5+ subset and the HLO1-6H5- subsets (*p* = 0.0067) (Figure 3C) (n = 5). ICC of bronchospheres after 42 days of culture showed that HLO1-6H5+ basal cells were able to differentiate and generate colonies containing basal cells (KRT5+ and KRT14+), goblet cells (MUC5B+), and ciliated cells (TUBA4A+) (Figure 3D). In summary, the HLO1-6H5 target marker identifies a subset of basal cells in primary hBECs that is enriched for bipotent, clonogenic cells.

### 2.5. The HLO1-6H5 Target Marker Subdivides ITGA6+ Basal Cells and Is Distinct from NGFR+ Basal Cells

**Figure 4:**
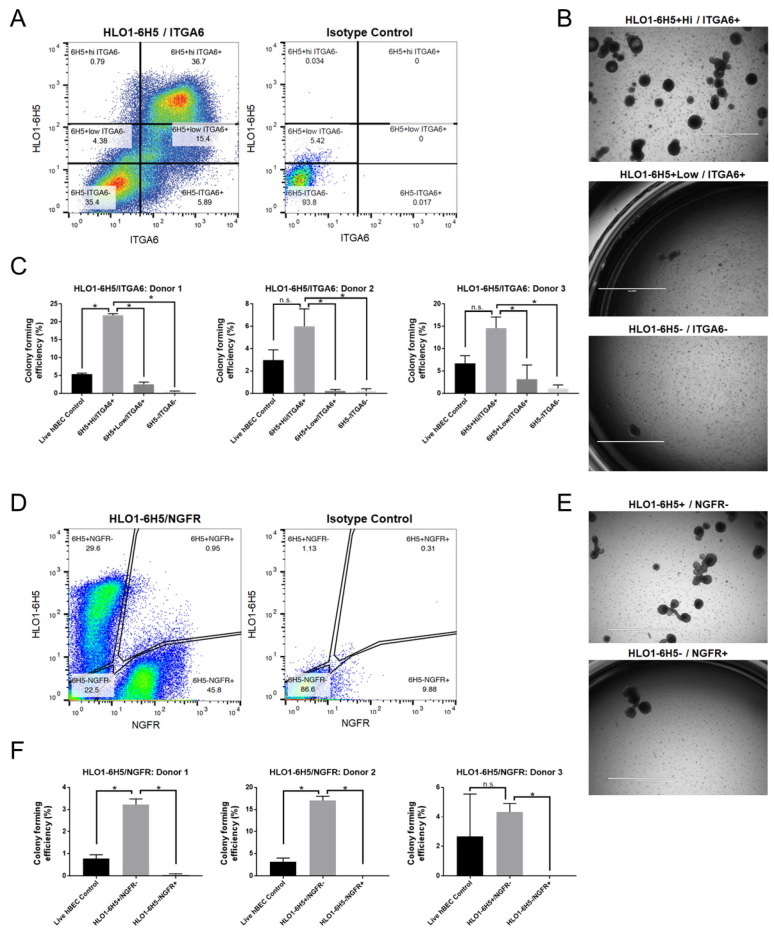
HLO1-6H5+ basal cells are a subset of ITGA6+ (integrin alpha 6) airway epithelial cells and are distinct from NGFR+ (nerve growth factor receptor) basal cells in uncultured primary hBECs. (**A**) Representative flow cytometry plots of HLO1-6H5 combined with ITGA6 to FACS-isolate cell subsets for assessment of clonogenicity in the bronchosphere assay. (n = three donor samples) (**B**) At Day 14, colonies were imaged and counted to determine which sorted subsets were enriched for clonogenic cells. (**C**) Graphs of colony-forming efficiency of each HLO1-6H5/ITGA6 cell subset across three donors, three technical replicates per donor. (**D**) Representative flow cytometry plots of HLO1-6H5 combined with NGFR to FACS-isolate cell subsets for assessment of clonogenicity in the bronchosphere assay. (n = three donor samples). (**E**) At Day 14, colonies were imaged and counted to determine which sorted subsets were enriched for clonogenic cells. (**F**) Graphs of colony-forming efficiency of each HLO1-6H5/NGFR cell subset across three donors, three technical replicates per donor. Paired *t*-test was performed for each donor sample, * *p* < 0.05. n.s. = not statistically significant. Data presented as mean (±S.D.). Scale bar: 2000 µm.

Rock et al. previously described two cell-surface markers for basal cells, NGFR and ITGA6 [27]. Both markers, along with HLO1-6H5, are expressed in uncultured hBECs and hBECs cultured according to the conditional reprogramming method (Figure S2) [28,29,30,31,32]. Two marker panels of HLO1-6H5 with NGFR or ITGA6 were used to FACS-isolate subsets of basal cells from uncultured primary hBECs. The HLO1-6H5 and ITGA6 panel showed that ITGA6 stained over 50% of the cells (Figure 4A).

HLO1-6H5 also stained over 50% of the cells, but the pattern of staining differed from ITGA6. HLO1-6H5 subdivided ITGA6+ cells into two subsets: HLO1-6H5+Low/ITGA6+ and HLO1-6H5+Hi/ITGA6+. The two subsets and the double negative population were FACS-purified into the bronchosphere assay and showed the HLO1-6H5+Hi/ITGA6+ to be highly enriched for clonogenic cells (Figure 4B) (n = 3). Three donor samples showed significant enrichment for clonogenic cells in the HLO1-6H5+Hi/ITGA6+ subset over the HLO1-6H5+Low/ITGA6+ and HLO1-6H5-/ITGA6- subsets, with average CFE values of 14.1%, 2.0%, and 0.58%, respectively, for each subset (*p* < 0.05) (Figure 4C).

The HLO1-6H5 and NGFR marker panel revealed two phenotypically distinct basal cell populations in uncultured primary hBECs (Figure 4D). There was no evidence of HLO1-6H5+/NGFR+ cells in three donor samples. The distinct basal cells were sorted and assessed for CFE differences via the bronchosphere assay (Figure 4E). The bronchosphere assay showed the HLO1-6H5+/NGFR- subset to be highly enriched for clonogenic cells with a CFE > 3.3% across three donors (Figure 4F). Meanwhile, the HLO1-6H5-/NGFR+ subset was severely depleted of clonogenic cells with a CFE < 0.01%, and in most wells, no colonies formed. Although both the HLO1-6H5 and NGFR antibodies target basal cells, there is a clear difference in the stem cell capabilities of these distinct subsets from uncultured hBECs. In summary, the HLO1-6H5+ basal cell subset is distinct from NGFR+ basal cells in terms of stem cell enrichment within primary hBECs. Meanwhile, the ITGA6 marker can be optimized by pairing with HLO1-6H5 in a marker panel to enrich a population of basal stem cells from primary hBECs.

### 2.6. The HLO1-6H5 Antibody Targets a Glycosylated Transferrin Receptor (TFRC) Proteoform

**Figure 5:**
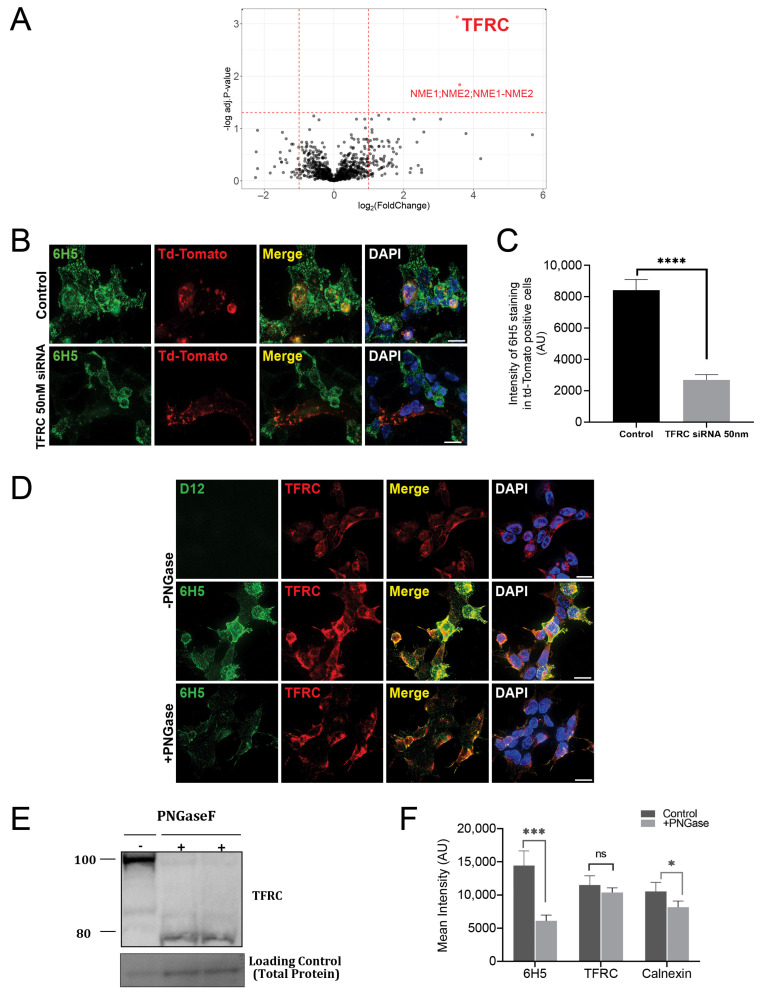
The HLO1-6H5 antibody recognizes a glycosylated transferrin receptor (TFRC) protein. (**A**) Volcano plot comparing the difference in enrichment between the HLO1-6H5 mAb and isotype control in the ASB experiment. Proteins significantly enriched by the HLO1-6H5 mAb over the control (adjusted *p*-value < 0.05, log2 fold-change > 3.5) are highlighted in red. Number of independent replicates = 3. (**B**) Immunocytochemistry with HLO1-6H5 mAb (green) of HLO1-6H5+ HEK293 cells treated with control non-targeting siRNA, TFRC siRNA, and pcNDA3.1-myr-tdTomato to show HLO1-6H5 staining after TFRC knockdown (bottom panel) versus control (top panel). Note, cells positive for tdTomato indicate cells that receive siRNA as well. Scale bar = 20 μm. (**C**) Quantitation of intensity of HLO1-6H5 staining in cells positive for td-Tomato in both control and TFRC knocked down cells using multiple unpaired *t*-test where *p* < 0.0001 ****. (Number of independent replicates = 4). (**D**) Immunocytochemistry of HLO1-6H5+ HEK293 cells with HLO1-6H5 mAb (green), an isotype control (green), and commercially available anti-TFRC (red) to show HLO1-6H5 staining intensity and colocalization with TFRC in the absence (top two rows) and presence (bottom row) of PNGase F treatment. Scale bar = 20 µm. (**E**) Western blot analysis of PNGase-treated cell lysates to visualize shifts in TFRC band compared to untreated control. (**F**) Quantification of immunostaining intensities for HLO1-6H5, TFRC, and Calnexin (+ve control for PNGase F) in untreated and PNGase F treated cells using multiple unpaired *t*-test where *p* < 0.001 *** for HLO1-6H5 and *p* < 0.05 * for the positive control. Number of independent replicates = 2.

To identify the cell surface target of HLO1-6H5, an HLO1-6H5-reactive HEK293 subpopulation was enriched and expanded using standard FACS and cell culture methods. Following a workflow previously described for identifying cell surface markers, we subjected this reactive HEK293 cell subpopulation to aminooxy-sulfhydryl-biotin (ASB) crosslinking using HLO1-6H5 as ligand, and resulting protein complexes isolated by affinity purification were subsequently interrogated via shotgun tandem mass spectrometry (MS) (n = 3) [33,34]. Results obtained with HLO1-6H5 were contrasted with those obtained using D12, an isotype control IgM antibody, where differential enrichment analysis indicated the most likely candidate for the HLO1-6H5 surface target was either TFRC or nucleoside diphosphate kinase (NME), as evidenced by a log2 fold change > 3.5 when compared to isotype-control labeled cells (adjusted *p*-value < 0.05) (Figure 5A).

NME proteins were de-prioritized as candidates since these proteins are known to catalyze nucleoside metabolic reactions and localize predominantly to the cytoplasm and nucleus [35]. TFRC/CD71, the top statistical protein target candidate from our crosslinking-MS analysis and a well-known cell surface receptor, was suspected as the most likely HLO1-6H5 target.

We confirmed TFRC as the HLO1-6H5 target using a siRNA knockdown approach. Due to a relatively low transfection efficiency observed for the reactive HEK293 cells, we incorporated a reporter (pcNDA3.1-myr-tdTomato) to mark transfected cells. Cells marked as transfected and, thus, subjected to TFRC-targeted siRNA knockdown indicated significantly reduced HLO1-6H5 staining (Figure 5B; n = 4 independent replicates, *p* < 0.0001 for 2-fold intensity decrease, Figure 5C). Quantitation of HLO1-6H5 mAb staining in the Td-Tomato positive cells showed a >2-fold decrease in staining intensity (*p* < 0.0001) (Figure 5C). Together, with our crosslinking/mass spectrometry analysis, these knockdown data indicate TFRC is the HLO1-6H5 target.

Co-immunostaining of reactive HEK293s indicated highly overlapping staining patterns for HLO1-6H5 and a commercial anti-TFRC antibody, but not for an IgM isotype control antibody D12 (top two rows of Figure 5D). In contrast, similar co-staining with HLO1-6H5 and commercial anti-NME1/2 antibodies failed to generate overlapping staining patterns. HLO1-6H5/TFRC co-localization patterns were highly overlapping but not completely congruent, suggesting the possibility that HLO1-6H5 recognizes a subset of TFRC proteoforms. TFRC has long been shown to be a glycosylated protein [36]. PNGase F pre-treatment of reactive HEK293s markedly and specifically diminished HLO1-6H5 staining, suggesting that the HLO1-6H5 mAb targets a glycosylated protein (Figure 5D). Western blots of TFRC in reactive HEK293 cell lysates showed that PNGase F treatment caused a discernible size shift of TFRC from 100 to <80 kDa, confirming that TFRC is a glycosylated protein (Figure 5E). Quantitative fluorescence analysis showed that PNGase treatment of reactive HEK293 cell lysates caused a significant reduction in staining intensity for HLO1-6H5 but did not impact TFRC staining, further confirming that the HLO1-6H5 mAb targeted a glycosylated protein, specifically TFRC (*p* < 0.001) (Figure 5F). In summary, our composite data indicate that the HLO1-6H5 mAb targets a glycosylated proteoform of TFRC.

## 3. Discussion

The basal epithelial cell compartment has long been established as a major source of adult stem/progenitor cells for the maintenance of the airway respiratory epithelium. The novel HLO1-6H5 mAb allowed us to explore the heterogeneity within the basal cell compartment of the respiratory epithelium as HLO1-6H5 identifies a subset of KRT5+ basal cells. In airway tissue, section HLO1-6H5 co-stains nearly all of the KRT5+ basal cells. Flow cytometry with HLO1-6H5 on uncultured hBECs showed that HLO1-6H5 targeted a 56–76% subset of KRT5+ basal cells (Figure 1 and Figure S2). The HLO1-6H5+ subset of uncultured hBECs exhibited functional properties that are characteristic of stem cells.

HLO1-6H5+ cells have the ability to differentiate, which the bronchosphere assay data confirmed with the emergence of ciliated and secretory cells from HLO1-6H5+ input cells. Thus, the HLO1-6H5+ subset likely represents an earlier-stage basal cell that has not yet been lineage committed (Figure 3D). Sectioned bronchospheres tend to have, at minimum, two layers of cells forming the wall of the structure. Most cells in bronchospheres are KRT5+ and KRT14+, suggesting a mitotically active basal cell phenotype. However, only a subset of the bronchosphere cells express both HLO1-6H5+ and KRT5+, and these cells comprise the outer layer of the structure (Figure 1C). This structural localization of HLO-6H5+ cells in bronchospheres mimics the physiological morphology of respiratory epithelium, in which the non-lineage committed basal stem cells reside along the basement membrane, away from the airway lumen. This evidence further supports the concept that the HLO1-6H5+ population is a subset of basal epithelial cells that are not lineage committed.

Bronchospheres are an ideal in vitro assay and model system for studying stem cells of the respiratory system and for mimicking the physiological environment or niche in which a target cell type resides. Currently, there are multiple bronchosphere protocols, some of which require stromal cell co-culture to generate colonies hBECs, and some that do not. Some pulmonary researchers have identified stromal cells as a means of optimizing the bronchosphere assay for assessing primary mouse stem cells [37,38]. Conversely, there are several studies that evaluated culture-expanded primary tissue (for a minimum of one passage) that did not require support cells to generate bronchospheres [5,39,40,41]. There was even one study that generated bronchospheres from uncultured hBECs without stromal cells by using hyperplastic airway tissue [42]. We optimized the assay to meet our needs of assessing stem cells from uncultured primary hBECs by adding human MRC5 stromal cells in co-culture. Our data show that the stromal cells are necessary for consistent bronchosphere formation from uncultured primary hBECs (Figure 2B,C). In summary, we have confirmed that a bronchosphere assay supplemented with MRC stromal cells can consistently evaluate stemness in uncultured primary airway epithelial cell populations isolated by cell surface markers.

Currently, the most well-established surface markers for isolating basal cells are NGFR and ITGA6 [3,4,6,14,42,43]. NGFR and ITGA6 are used for isolating bulk basal cells from primary and culture-expanded airway epithelial cells, but the HLO1-6H5 mAb is optimized for isolating a clonogenic basal cell subset from uncultured primary hBECs. Our data show that the HLO1-6H5 target marker is distinct from NGFR, and that each marker identifies a discrete basal cell population. The distinction is further magnified in the bronchosphere assay as all the clonogenic cells are enriched in the HLO1-6H5+ basal cell subset, not the NGFR+ basal cells. When paired with ITGA6, HLO1-6H5 is co-expressed with ITGA6 and allows for the resolution of HLO1-6H5+Hi and low populations within dispersed primary human airway cells. These two subsets, HLO1-6H5+Hi/ITGA6+ and HLO1-6H5+Low/ITGA6+, are functionally distinct as the HLO1-6H5+Hi subset is the most enriched for clonogenic cells compared to the HLO1-6H5+Low subset. Based on the work shown here, we propose a model of basal cell heterogeneity in which there are three subsets of basal cells: HLO1-6H5-/ITGA6-/NGFR+, HLO1-6H5+Low/ITGA6+/NGFR-, and HLO1-6H5+Hi/ITGA6+/NGFR-, of which the HLO1-6H5+Hi basal cell subset is enriched for self-renewing, bipotent stem/progenitor cells.

The antigen target of the HLO1-6H5 mAb is glycosylated TFRC/CD71, a cell surface protein involved in receptor-mediated iron transport into the cell via endocytosis. TFRC’s functional role of transporting iron into cells has been shown to impact cellular proliferation, a stem cell attribute, as TFRC expression is increased in proliferating cells in the liver and intestines, with one study showing that the reduction of proliferation in TFRC-intestinal epithelial cells can be rescued by TFRC expression [44]. The iron transport function of TFRC also has been shown to impact hematopoietic stem cell differentiation and proliferation, as both cellular processes require cellular iron [45]. The cell surface expression of TFRC is impacted by glycosylation, as studies have shown that cell surface localization of TFRC was reduced in TFRC mutants that lacked specific glycosylation sites and rescued in TFRC mutants that regained a glycosylation site [46,47]. TFRC has been used as a marker to identify and isolate human epithelial or mesenchymal stem cells in bone marrow, periodontal ligament, umbilical cord blood, adipose, and hair follicles. Also, of note, it was able to isolate basal cell subsets from eye and skin that are enriched for stemness in colony-formation assays [48,49,50]. In respiratory tissue, TFRC has not previously been used to identify and isolate epithelial stem cells or other cell subsets. The identification of a TFRC proteoform as a cell surface marker for stem cells in the basal cell compartment of human respiratory epithelium provides another tool for studies of lung cell populations and their respective roles in development, regeneration, and respiratory disease pathology. Additionally, this work raises questions of the functional significance of a TFRC proteoform as it relates to airway epithelial cell differentiation and proliferation.

The potential utility for the HLO1-6H5 target cell surface marker extends into both research and translational applications. In terms of research utility and capabilities, the HLO1-6H5 target marker will enable study efforts to further subdivide the basal cell population into subsets that are phenotypically, functionally, and transcriptionally distinct. This focus on basal cell heterogeneity addresses questions regarding the specific cells that are responsible for airway homeostasis and epithelial repair; this mAb could address those questions by isolating a novel subset of KRT5+ human basal cells. This novel basal cell subset is also a potential target for ex vivo expansion of donor tissue for lung tissue engineering or cell-based therapeutics that require a population enriched for stem/progenitor cells.

The HLO1-6H5 target marker is also potentially useful for studies of respiratory disorders due to its potential to effectively isolate a novel cell subset from respiratory tissue affected by conditions such as cystic fibrosis (CF), chronic obstructive pulmonary disorder (COPD), and idiopathic pulmonary fibrosis (IPF) to elucidate the role of a subset of basal or basal-like cells in the pathogenesis of these diseases. Basal cell hyperplasia is a characteristic of both COPD and CF pathogenesis [51]. IPF has been shown to exhibit an increased distribution of basal cell marker expression in distal airways due to the presence of aberrant cells that express both alveolar cell and basal cell markers; the aberrant cells are potential drivers of IPF progression [51,52]. The isolated primary cells could also be used to develop additional in vivo and in vitro models to supplement existing study models of each disease, similar to the bronchosphere system employed in this body of work [20,51,52].

Another potential translational application of the HLO1-6H5+ basal cell subset and TFRC proteoform is in the context of cancer. The subset and proteoform could be relevant diagnostic markers or therapeutic targets for lung cancers that involve an epithelial basal stem/progenitor population. A suspected driver of lung squamous cell cancer (SCC) is basal cells, as a primary characteristic of lung SCC is basal cell metaplasia and increased expression of canonical basal cell markers like KRT5, SRY-box 2 (SOX2), and tumor protein p63 [53,54]. Furthermore, our findings may benefit studies across a broad range of oncological indications due to the evidence that TFRC has been identified as a candidate therapeutic target because of its elevated expression in several types of cancer: brain, liver, breast, colon, ovarian, prostate, leukemia, and lung, with a push towards developing anti-TFRC antibodies to facilitate the delivery of anti-cancer drug conjugates, or for the disruption of TFRC function and, thereby, cancer progression [55,56]. Shapiro et al. used a novel mAb to identify a glycosylated TFRC that was overexpressed in colon cancer but absent in paired healthy colon tissue [57]. Recent in vitro studies have shown that increased expression of TFRC reduces non-small cell lung cancer (NSCLC) cell proliferation, demonstrating early potential for TFRC as a therapeutic target [58,59]. As a marker, recently, *TFRC* was discovered as part of a gene signature that could predict overall survival rates in patients with lung SCC [60]. The *TFRC* gene has also been deemed useful in genetic panels that provide either early detection or prognosis for lung adenocarcinoma, another NSCLC like SCC [61,62]. In summary, our generation and characterization of the novel HLO1-6H5 monoclonal antibody, its target surface marker of TFRC, and the lung progenitor cell subset that it isolates will provide opportunities to further understand normal lung cell populations and their respective functions and also potentially advance our knowledge of the mechanisms underlying multiple respiratory conditions and cancers of the lung and other tissue systems.

## 4. Material and Methods

### 4.1. Human Bronchial Epithelial Cell (hBEC) Isolation

Healthy (no diagnosis of respiratory disease) non-smoker donor lungs were acquired by the UNC Chapel Hill Marisco Lung Institute’s Cystic Fibrosis Center Tissue Procurement and Cell Culture Core (Table S1). Tracheal–bronchial airway segments disassociated with 0.1% protease XIV (Sigma #P5147, Saint Louis, MO, USA) and 0.001% DNase (Sigma #DN25) were then scraped with a scalpel to yield dispersed viable hBECs [63]. Isolated hBECs were resuspended in F12 media (ThermoFisher #11765, Waltham, MA, USA) and shipped overnight on ice to OHSU.

### 4.2. Organoid Culture of Human Bronchial Epithelial Cells

Isolated hBECs were grown under the organoid conditions developed by the Hans Clevers Laboratory for pancreatic and liver organoids [25]. Primary hBECs were embedded in >95% Matrigel^®^ extracellular matrix (Corning #356231, Corning, MA, USA) and submerged in growth media containing ALK5 inhibitor SB431542 (Tocris #1614, Minneapolis, MN, USA) and supplemented with WNT-agonist R-spondin, Noggin, and FGF-10 [25]. Intact hBEC-derived organoids were harvested after 7 days of culture in standard 5% CO_2_ and 37 °C incubator conditions when lumen formation was evident.

### 4.3. Monoclonal Antibody (mAb) Production

Mice were immunized with lung organoids grown from primary hBECs according to the previously mentioned protocol [24,26,64]. Mice were sacrificed 4 days after the final immunization to harvest splenocytes for fusion with SP2/0 Ag14 myeloma cells and growth in methylcellulose-containing hypoxanthine-aminopterin-thymidine medium to selectively expand hybridoma clones. The mAb-containing media of each clone were screened by immunohistochemistry on acetone-fixed human airway tissue and by flow cytometry on hBECs. Clones of interest were further culture-expanded to generate additional mAb and cryopreserved in Dulbecco’s Modified Eagle Medium (DMEM) (ThermoFisher # 11965-092) with 10% fetal bovine serum (FBS) (Hyclone #SH30071.03, Logan, UT, USA) and 10% dimethylsulfoxide (DMSO). mAb isotyping was done with a mouse immunoglobulin isotyping enzyme-linked immunosorbent assay kit (BD Biosciences # 550487, Franklin Lakes, NJ, USA) and by flow cytometry using isotype-specific fluorescent goat anti-mouse secondary antibodies (Jackson ImmunoResearch, West Grove, PA, USA).

### 4.4. Human Airway Tissue Sections

Fresh human lung tissue from healthy donor airways was frozen in OCT (Optimal Cutting Temperature) compound blocks at the UNC Chapel Hill Marisco Lung Institute’s Cystic Fibrosis Center Tissue Procurement and Cell Culture Core under IRB-approved protocols (Office of Human Research Ethics/IRB study #03-1396). The core staff dissected trachea and bronchi away from connective tissue and lymph nodes of cadaveric lungs. Freshly dissected airways were cut into segments and embedded and frozen in OCT compound. Airway tissue sections (5–7 µm) were cut using a Reichert 2800 Frigocut cryostat (Reichert Scientific Instruments, Depew, NY, USA). Tissue sections were fixed in acetone for 5 min at −20 °C, air-dried at room temperature, and then stored under dry conditions at −80 °C for up to 6 months.

### 4.5. Fluorescent Immunohistochemistry (IHC) and Immunocytochemistry (ICC)

Acetone-fixed tissue sections from fresh, healthy donor lungs were blocked with 5% FBS and 5% goat serum (ThermoFisher #PCN5000) in Permeabilization Buffer (eBioscience #88-8824-00, Waltham, MA, USA), incubated with primary antibodies for 30 min, and washed 3× with phosphate-buffered saline (PBS). Primaries were detected with fluorescent secondary antibodies for 30 min, PBS washed 3×, with the second wash containing Hoechst 33342 (ThermoFisher #H3570) nuclear dye, then mounted with Fluormount G (Southern Biotech #0100-01, Birmingham, AL, USA). Staining buffer was Permeabilization Buffer (eBioscience) with 5% FBS. Stained sections were imaged on a Zeiss Axioskop 2 plus (Zeiss, Group, Oberkochen, Germany) microscope. Primary antibodies included: HLO1-6H5 mAb, polyclonal rabbit anti-human KRT5 (BioLegend #905501, San Diego, CA, USA), rabbit anti-human KRT14 (ThermoFisher #MA5-16370), rabbit anti-human acetylated α-tubulin (Cell Signaling Technology #5335, Danvers, MA, USA), rabbit anti-human MUC5B (Santa Cruz #SC-20119, Dallas, TX, USA), or rat anti-human KRT8/18 (Developmental Studies Hybridoma Bank #TROMA-I-C, Iowa City, IA, USA). Secondary antibodies included: Cy3-goat anti-mouse IgG (H + L) (EMD Millipore #AP124C, Burlington, MA, USA) and DyLight488-goat anti-rabbit IgG (H + L) (ThermoFisher #35553).

For ICC, 3 × 10^5^ HEK293T cells were seeded on glass coverslips in six-well clusters, grown 24–48 h, fixed in 2% paraformaldehyde, and antibody stained for TFRC (ThermoFisher #13-6800) and/or the HLO-6H5 mAb and its isotype. Secondaries were: Alexa 488- and 594- anti-mouse and anti-rabbit antibodies (Jackson ImmunoResearch, West Grove, PA, USA). Stained cells were DAPI (4′,6-diamidino-2-phenylindole) counterstained, then mounted on glass slides in SlowFade antifade medium (Invitrogen, Waltham, MA, USA). Images were acquired on a Leica Thunder microscope (Leica Microsystems, Wetzel, Germany) and analyzed with image-processing software (Volocity, Version 6.5, Quorum Technologies, Puslinch, ON, Canada).

### 4.6. Flow Cytometry/FACS (Fluorescence-Activated Cell Sorting)

Staining and wash buffer were cold RPMI media (Gibco # 11875-093, Waltham, MA, USA) with 5% FBS. For flow cytometry and FACS, cells were incubated with primary antibodies for 30 min at 4 °C, washed 2×, detected with fluorescent secondary antibodies for 30 min at 4 °C, washed 2×, and viability stained with 2 µg/mL propidium iodide. Primaries were: HLO1-6H5 mAb, mouse anti-human NGFR (EMD Millipore #05-446), and rat anti-human ITGA6 (BioLegend #313602). Secondaries (from Jackson ImmunoResearch) were: PE-goat anti-mouse IgM secondary F(ab’)_2_ fragment (#115-116-075), A488-goat anti-mouse IgG secondary antibody (#115-545-164), APC-donkey anti-rat secondary F(ab’)_2_ fragment (#712-136-153), and DyLight488-goat anti-rabbit IgG (H + L).

Intracellular flow cytometry followed above protocol with added steps: 30 min in e450 Fixable Viability dye (eBioscience #65-0863-14) at 4 °C before staining with primary cell-surface antibodies, 30 min in IC Fixation Buffer (4% paraformaldehyde) (eBioscience #88-8824-00) at 4 °C after secondaries detection for cell surface antibodies, then 30 min in eBioscience Permeabilization Buffer at 4 °C, then 30 min staining for intracellular markers KRT5 and KRT14 (rabbit anti-human) at 4 °C, wash 2× with Permeabilization buffer, and then DyLight488-goat anti-rabbit IgG (H + L) secondary. e450 Viability dye (eBioscience #65-0863-14) substituted for propidium iodide.

Cells analyzed on BD Biosciences’ LSRII flow cytometer or Influx Cell Sorter with a 140 µm nozzle after live/dead exclusion and doublet discrimination.

### 4.7. Cell Culture

HEK293T cell line was grown in DMEM (Gibco, Carlsbad, CA, USA) supplemented with 10% FBS in an atmosphere of 5%CO_2_/95% air. Stock cultures were maintained weekly by seeding 5 × 10^5^ cells per 100 mm dish in 10 mL of complete culture medium with a medium change on Day 3 or 4. New stocks are initiated after 15 passages.

### 4.8. Crosslinker Reagent Synthesis and Validation

ASB crosslinker reagent was synthesized by the OHSU Medicinal Chemistry Core. Reagent potency was verified in biological assays using a characterized anti-Epcam (epithelial cell adhesion molecule) antibody and an isotype control in two cell lines known to express surface Epcam. Mass spectrometry (MS) analysis indicated Epcam as the top target in both cell lines, and a greater fold change versus isotype control was detected for the cell line known to exhibit relatively higher Epcam expression.

### 4.9. Aminooxy-Sulfhydryl-Biotin (ASB) Crosslinking and Mass Spectrometry (MS) Analysis

ASB crosslinking MS assays to detect HLO1-6H5’s target antigen used HLO1-6H5 mAb, an IgM isotype control antibody, and HLO1-6H5 reactive HEK293 cells selected by FACS. PEG4-SPDP (ThermoScientific) was employed as initial amine-to-sulfhydryl pyridylthiol-based crosslinker [33,34]. Resulting peptides were analyzed by label-free tandem MS on a Q-Exactive High Field Orbitrap mass spectrometry instrument (ThermoScientific) coupled to an UltiMate 3000 RSLCnano (ThermoScientific, Waltham, MA, USA) liquid chromatograph instrument in the OHSU Proteomics Shared Resource Core Facility using standard methods for data-dependent acquisition. Raw MS data files searched against the UniProt human proteome database from 18 July 2018, using MaxQuant v1.6.2.2 with standard settings and LFQ enabled [65,66]. Oxidation (M) and acetylation (N-term) were set as fixed modifications and carbidomethyl (C) as variables. The FDR (false discovery rate) at the protein and PSM (peptide-spectrum match) levels were set to 0.01. Match between runs (MBR) was enabled. The resulting output data were further processed and analyzed in R (version 3.6.0) using MSstats (3.16.0) and R packages dplyr (0.8.0.1) and ggplot2 (3.1.1) [67,68].

### 4.10. PNGase F Treatment

At 24 h after cell seeding, HLO1-6H5 reactive HEK293T cells (3 × 10^5^) were treated with PNGase F (50 U/mL, NEB BioLabs, Ipswich, MA, USA) or vehicle in serum-free media for 4–6 h. Western blotting by standard procedures assessed TFRC migration shift as indicative of glycan removal. Cell staining following treatment was performed as described above.

### 4.11. siRNA Knockdown

HLO1-6H5 reactive HEK293T cells were seeded at 3 × 10^5^ cells in a six-well cluster for 24 h, then transfected with 50 nm TFRC targeting and control SMARTpool siRNAs along with 0.5 μg of pcNDA3.1-myr-tdTomato using Dharmafect1 reagent for 72 h according to manufacturer’s instructions (GE Healthcare Dharmacon, Inc., Lafayette, CO, USA). ICC was described above, and Western blotting employed standard procedures to assess knockdown efficiency.

### 4.12. Bronchosphere Assay

hBECs were FACS isolated into plates containing Transwell^®^ inserts (Sigma #Z354988, Saint Louis, MO, USA) pre-coated with a 1:1 mixture of MatriGel (Corning #356231) and air–liquid interface media containing 24,000 MRC5 human fetal lung stromal cells [63]. Cultures grew in 5% CO_2_, 37 °C incubator conditions for 2 weeks, and then colonies were counted.

Colonies were harvested at 6 weeks with Corning Cell Recovery Solution (#354253) for 1 hr at 4 °C. Released colonies were embedded in optimal cutting temperature (OCT) compound and stored at −80 °C. Bronchospheres in OCT compound were cryosectioned (7 µm), acetone fixed for 5 min at −20 °C, air-dried, and stored dry for up to 6 months at −80 °C.

### 4.13. Statistical Analyses

All data are presented as the mean ±SD. All colony formation efficiency results were statistically analyzed by paired *t*-tests with GraphPad Prism v8.01 (GraphPad Software, San Diego, CA, USA). Quantification of fluorescent intensity of immunohistochemical staining and Western blot immunostaining were analyzed by multiple unpaired *t*-tests. *p*-value < 0.05 was considered statistically significant.

MS data were statistically analyzed in R using MSstats and R packages dplyr and ggplot2 [67,68].

## Data Availability

Raw MS data were deposited to MassIVE on 3 September 2024 and are accessible at ftp://massive.ucsd.edu/v08/MSV000095761/. Other data will be made available on request.

## Supplementary Materials

The supporting information can be downloaded at: https://www.mdpi.com/article/10.3390/ijms25189863/s1.
