# Supplementary material for: Identification of a Novel Subset of Human Airway Epithelial Basal Stem Cells
Source: Int J Mol Sci. 2024 Sep 12;25(18):9863. doi: 10.3390/ijms25189863 (PMC11432080; doi:10.3390/ijms25189863)
Supplement: Supplementary file 1 [file ijms-25-09863-s001.zip › ijms-3186906-supplementary.pdf]

Supplementary Materials

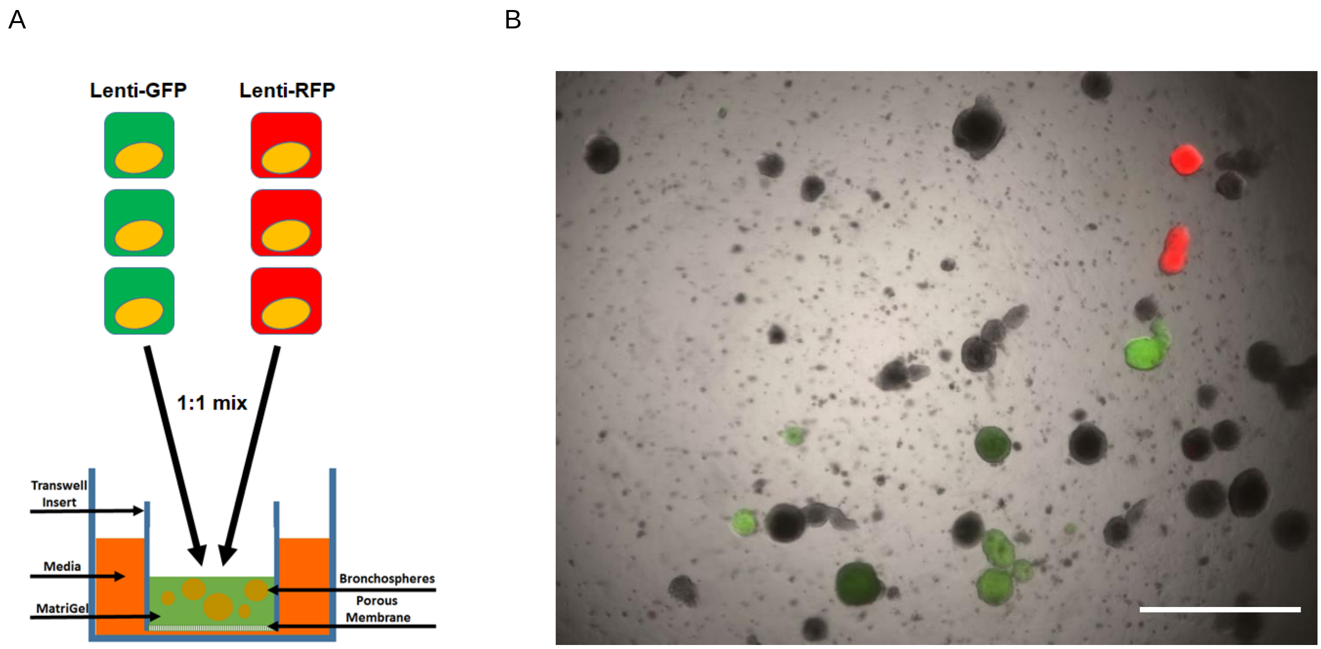

Figure S1. Bronchospheres are clonogenic and derived from single cells, Related to Figure 2. A) Primary hBECs transduced with either GFP or dsRed were seeded into the bronchosphere assay at a 1:1 GFP to dsRed ratio and grown for 14 days to generate bronchospheres in co-culture with MRC5 human fetal lung fibroblast; B) Representative image of bronchospheres grown for 14 days from unsorted hBECs that are unlabeled or that express GFP or dsRed (Number of independent replicates = 3). Scale bar: 2000 μm.

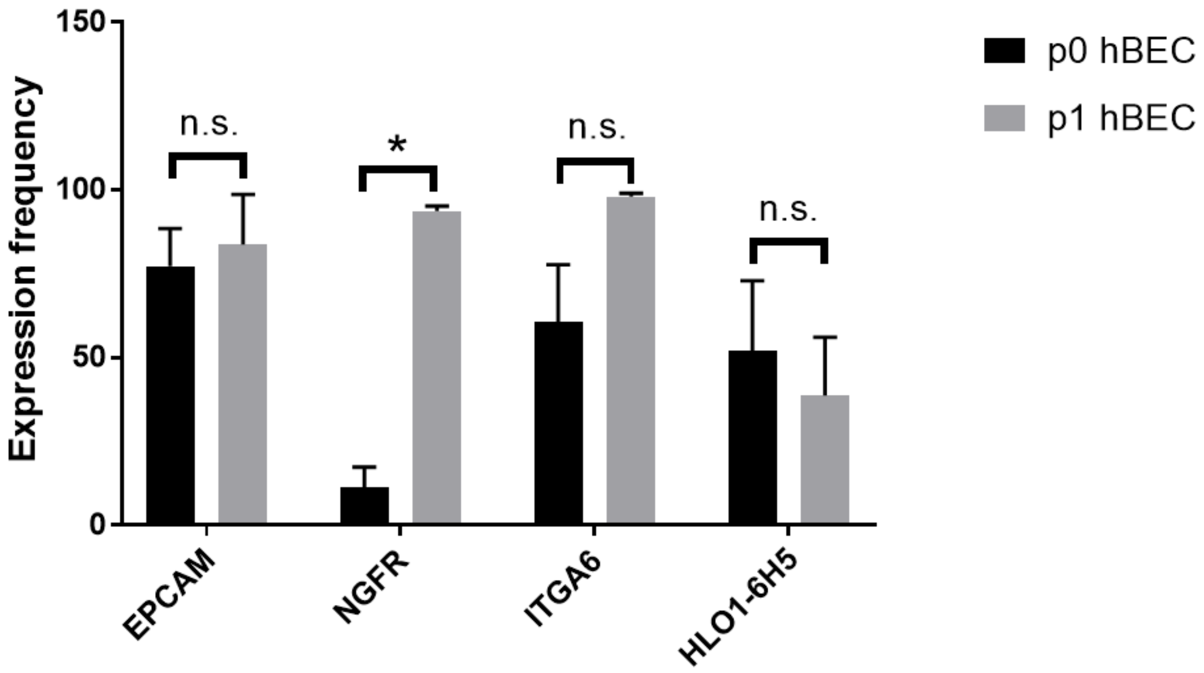

Figure S2. Expression of cell surface markers from uncultured primary hBECs to passage 1 hBECs grown under conditional reprogramming culture conditions, Related to Figure 4.
A comparison of expression frequency of the HLO1-6H5, NGFR, ITGA6, and EPCAM cell surface markers in donor matched passage 0 and passage 1 hBECs grown on irradiated mouse 3T3-j2 embryonic fibroblast feeder cells with Rho kinase inhibitor according to the method known as conditional reprogrammed cell culture. (n = 3 donor samples) Paired t-test, *p<0.05. n.s. = not statistically significant. Data presented as mean (+/- S.D.).

Table S1. Demographics of lung tissue donors, Related to Figures 1 through 4.
Uncultured primary hBECs were sourced from non-smoker donors without any prior diagnosis of respiratory disease.

Donor Number	Age (years)	Sex	Race/ethnicity	Smoking status
1	18	M	Caucasian	Non-smoker
2	22	M	Caucasian	Non-smoker
3	48	F	Caucasian	Non-smoker
4	14	M	Caucasian	Non-smoker
5	53	F	Caucasian	Non-smoker
6	36	M	Hispanic	Non-smoker
7	54	F	Caucasian	Non-smoker
8	17	M	Hispanic	Non-smoker
9	40	M	Caucasian	Non-smoker
10	16	M	Caucasian	Non-smoker

11	67	M	Caucasian	Non-smoker

Table S2. Bronchospheres derive from single GFP+ or dsRed+ cells, Related to Figure S1. Spheroids that formed are either GFP-labelled, dsRed-labelled, or unlabeled. There were no colonies that were a mixture of GFP-and dsRed-labelling.

Replicate	# of Red Colonies	# of Green Colonies	# of Red & Green Colonies
1	14	22	0
2	8	13	0
3	8	23	0

Spheroids that formed are either GFP-labelled, dsRed-labelled, or unlabeled. There were no colonies that were a mixture of GFP-and dsRed-labelling.

Supplemental Experimental Procedures

GFP- and dsRed-labelling of human bronchial epithelial cells

Incubated primary hBECs at a density of 4000-5000 cells/µL for 4 hours at 37°C suspended in F12 media (ThermoFisher #11765) with 2 µg/mL polybrene and a 1:25 dilution of either GFP- or dsRed-lentiviral particles to achieve low efficiency transduction, thereby reducing the chance of bronchosphere colonies forming from two cells that were labelled with the same fluorescent protein. Added a 1:1 mixture of GFP- and RFP-labelled hBECs to the bronchosphere assay and grew under standard 5% CO2, 37°C incubator conditions for 2 weeks, The colonies that formed were counted and assessed for green fluorescent protein (GFP) and dsRed expression.

Conditional reprogramming cell culture

Embryonic mouse 3T3-j2 fibroblast feeder cells were irradiated to 22gy for mitotic arrest and frozen in aliquots for use. Three million irradiated 3T3-j2 feeders were seeded 2-24 hours in advance of hBEC seeding. A 30:1 hBEC to 3T3 feeder cell ratio was cultured in the presence of growth media with 5µmol/L Rho kinase inhibitor Y-27632 and 10ng/mL human EGF (Sigma #E9644) [1–3]. Cultures grew at 37°C and 5% CO2 in a humidified incubator for 1 week until harvest, when hBECs were ~90% confluent and most 3T3 feeder cells had detached from the plate surface. Harvested passage 1 hBECs were stained for flow cytometry according to the previously listed protocol.

Supplemental References

1. Suprynowicz, F.A.; Upadhyay, G.; Krawczyk, E.; Kramer, S.C.; Hebert, J.D.; Liu, X.; Yuan, H.; Cheluvaraju, C.; Clapp, P.W.; Boucher Jr., R.C.; et al. Conditionally Reprogrammed Cells Represent a Stem-like State of Adult Epithelial Cells. *Proc Natl Acad Sci U S A* **2012**, *109*, 20035–20040, doi:10.1073/pnas.1213241109.

2. Liu, X.; Krawczyk, E.; Suprynowicz, F.A.; Palechor-Ceron, N.; Yuan, H.; Dakic, A.; Simic, V.; Zheng, Y.; Sripadhan, P.; Chen, C.; et al. Conditional Reprogramming and Long-Term Expansion of Normal and Tumor Cells from Human Biospecimens. *Nat Protoc* **2017**, *12*, 439–451, doi:10.1038/nprot.2016.174.

3. Liu, X.; Ory, V.; Chapman, S.; Yuan, H.; Albanese, C.; Kallakury, B.; Timofeeva, O.A.; Nealon, C.; Dakic, A.; Simic, V.; et al. ROCK Inhibitor and Feeder Cells Induce the Conditional Reprogramming of Epithelial Cells. *AJPA* **2012**, *180*, 599–607, doi:10.1016/j.ajpath.2011.10.036.
